# Diffuse posterior leukoencephalopathy in MELAS without stroke-like episodes: A case report

**DOI:** 10.1097/MD.0000000000033725

**Published:** 2023-05-05

**Authors:** Peng Bai, Yinling Feng, Jin Chen, Hong Chang

**Affiliations:** a Department of Neurology, Inner Mongolia People’s Hospital, Hohhot, People’s Republic of China; b Inner Mongolia Medical University, Jinshan Development Zone, Hohhot, People’s Republic of China.

**Keywords:** case report, epilepsy seizures, leukoencephalopathy, MELAS syndrome, mitochondrial disorders

## Abstract

Mitochondrial encephalopathy, lactic acidosis, and stroke-like episodes (MELAS) is the most common subtype of mitochondrial encephalopathy. In the past, it was believed that most hereditary white matter lesions were lysosome storage disorders or peroxisome diseases. However, in recent years, white matter lesions have been increasingly regarded as a common feature of patients with mitochondrial diseases. In addition to stroke-like lesions, about half of the patients with MELAS reported white matter lesions in the brain.

**Patient concerns:**

Herein, we provide a case of A 48-year-old female who presented with episodic loss of consciousness with twitching of extremities. Previous medical history revealed 10 years of history of epilepsy, 10 years of history of diabetes, a history of hearing loss, and unknown etiology. Ancillary findings included brain magnetic fluid-attenuated inversion recovery showed symmetrical lesions in the bilateral parietal lobe with high signal intensity at the edge, and high signal intensity in the bilateral occipital lobe, paraventricular white matter, corona radiata, and the center of semiovale.

**Diagnoses:**

Mitochondrial deoxyribonucleic acid gene sequencing returned A3243G point mutation and it supports the diagnosis of intracranial hypertension.

**Interventions:**

Considered the diagnosis of symptomatic epilepsy, the patient was treated with mechanical ventilation, midazolam, and levetiracetam, and the limb twitching symptoms were controlled. The patient was comatose, chronically bedridden, with gastrointestinal dysfunction, and was treated prophylactically with antibiotics against infection, parenteral nutrition, and other supportive measures. B vitamins, vitamin C, vitamin E, coenzyme Q10, and idebenone were given, and mechanical ventilation and midazolam were stopped after 8 days. He was discharged from the hospital on 30 days and continued symptomatic treatment with B-vitamins, vitamin C, vitamin E, coenzyme Q10, and idebenone, and antiepileptic treatment with levetiracetam, with outpatient follow-up.

**Outcomes:**

No further seizures were recorded and the patient recovered well.

**Lessons:**

MELAS syndrome without stroke-like episodes of diffuse posterior cerebral white matter lesions is rare in clinical practice, and the possibility of MELAS syndrome should be considered in symmetric posterior cerebral white matter lesions.

## 1. Introduction

Mitochondrial encephalopathy, lactic acidosis and stroke-like episodes (MELAS) is the most common subtype of mitochondrial encephalopathy. Point mutations most commonly cause it at the A3243G of the transfer ribonucleic acid (tRNA) l-isoleucine gene in the mitochondrial deoxyribonucleic acid (mtDNA). This, in turn, affects mitochondrial protein synthesis and nutrient metabolism, leading to disease. The onset of illness is often in early adulthood. Symptoms include headache, vomiting, hemiplegia, blindness, and epileptic seizures. Patients generally have short stature and low intelligence.^[1,2]^ In the past, it was believed that most hereditary white matter lesions were lysosome storage disorders or peroxisome diseases. However, in recent years, white matter lesions have been increasingly regarded as a common feature of patients with mitochondrial diseases.^[3–5]^ In addition to stroke-like lesions, about half of the patients with MELAS reported white matter lesions in the brain.^[6]^ We report the clinical presentation, laboratory tests, and treatment of a patient with MELAS syndrome, a rare diffuse posterior cerebral white matter lesion without stroke-like episodes, and review the relevant literature.

## 2. Case presentation

**Figure 1.**
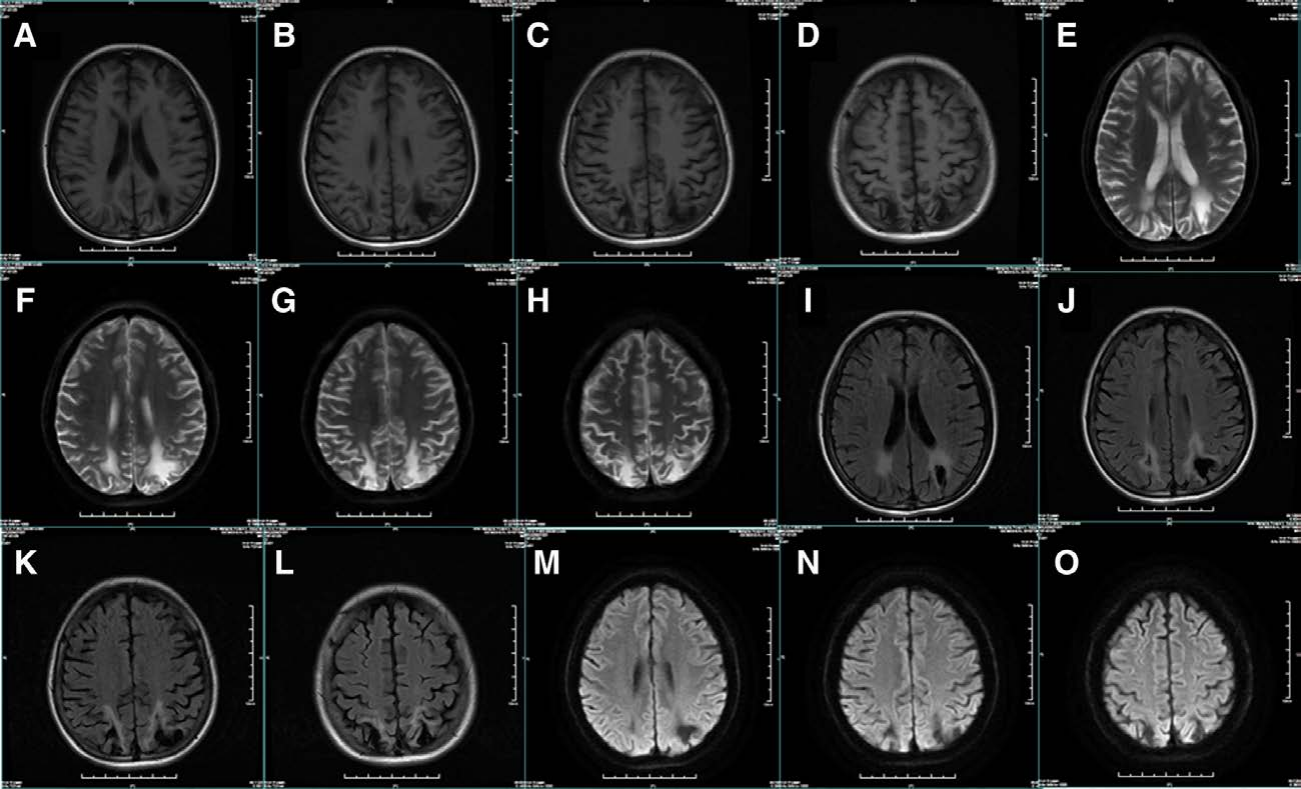
Magnetic resonance imaging of the patient.

**Figure 2.**
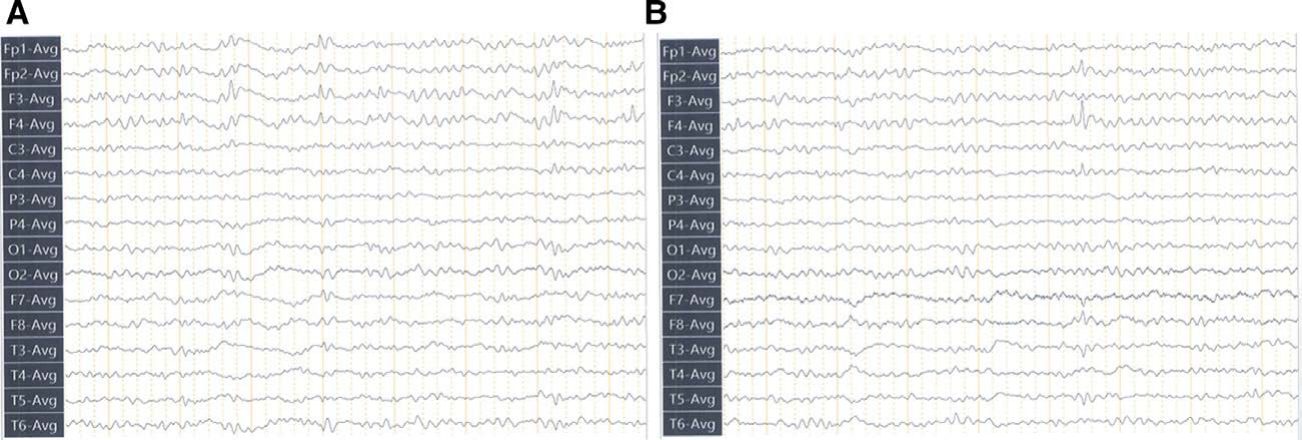
Electroencephalography of the patient.

A 48-year-old female was admitted to the authors’ department for episodic loss of consciousness with twitching of extremities for 5 hours. The patient was admitted to the hospital 5 hours ago; loss of consciousness occurred during mealtime, accompanied by limb twitching, limb tonicity, manifested by double eye hanging, double upper limb flexion, accompanied by lip cyanosis, no foaming at the mouth, diarrhea, no tongue bite, recurrent symptoms, consciousness did not return during the convulsions, cranial Computed tomography showed that the right temporal lobe deep hypodense foci, bilateral posterior horn of the ventricle, occipital, parietal cortex patchy hypodense shadow, considered persistent epilepsy. The patient was given mechanical ventilation, midazolam, and sodium valproate to control epilepsy, and dopamine treatment for low blood pressure. Physical examination revealed a sedative state, bilateral pupil equal circle, diameter about 2 mm, slow to light reflex, bilateral facial lines symmetrical, bilateral limb tendon reflex decreased, bilateral pathological signs negative, other physical examination did not cooperate. Previous medical history revealed 10 years of history of epilepsy, irregular oral drugs, 10 years of history of diabetes, irregular oral drugs, history of hearing loss, and unknown etiology. His brother died after being diagnosed with “epilepsy” at the age of 2, the cause of which is unknown. Brain magnetic fluid-attenuated inversion recovery showed symmetrical lesions in the bilateral parietal lobe with high signal intensity at the edge and high signal intensity in the bilateral occipital lobe, paraventricular white matter, corona radiata, and the center of semiovale (Fig. 1). Electroencephalography showed that 6 to 7 Hz low to medium amplitude theta waves and theta rhythm in the whole conduction in the quiet closed-eye state, interspersed with a small amount of low amplitude fast waves, poor left-right symmetry, poor regulation, and amplitude modulation, and incompatibility between open and closed eyes. In the monitoring, the whole conduction frequency was slowed down, and the low to medium wave amplitude sharp waves in the frontal poles and frontal leads emanated from the right frontal area asynchronously. No clinical seizures were observed during the monitoring (Fig. 2). The electromyography of the needle pole showed that there was no obvious myogenic or neurogenic electromyogram change in the examined muscle, and the conduction velocity of the bilateral median nerve, bilateral tibial nerve, and right common peroneal nerve slowed down, the sensory conduction sensory nerve action potential of bilateral median nerve was not elicited, the amplitude of sensory nerve action potential wave of sensory nerve decreased, the latency of F wave of the motor nerve was normal, the amplitude of F wave of tibial nerve decreased, and the latency of H reflex of the tibial nerve was normal. No abnormality was found in cardiac ultrasound and electrocardiogram. The pressure of the cerebrospinal fluid is normal, with cerebrospinal fluid leukocytes of 3 × 10^6^/L (reference range: <8 × 10^6^/L), an elevated lactic acid (8.30 mmol/L), a normal adenosine dehydrogenase (0.77 U/L), an normal protein (0.28 g/L), and normal glucose (2.96 mmol/L). Virus encephalitis was also initially considered because of the symptoms of epilepsy. However, the analysis of cerebrospinal fluid metagenomic next-generation sequencing did not detect virus sequence numbers. Her cerebrospinal fluid and serum oligoclonal bands are negative. The results of macroeconomic deoxyribonucleic acid (DNA) and RNA detection of pathogenic microorganisms in cerebrospinal fluid were negative. Autoimmune antibodies, including anti-N-methyl-D-aspartate, anti-glial fibrillary acidic protein, anti-α-amino-3-hydroxy-5-methyl-4-isoxazole propionic acid 1, anti-α-amino-3-hydroxy-5-methyl-4-isoxazole propionic acid 2, anti-gamma-aminobutyric acidβ, leucine-rich glioma-inactivated 1, anti-contactin-associated protein-like 2, anti-glutamic acid decarboxylase 65-kilodalton isoform were negative in cerebrospinal fluid and serum. Blood routine test showed that hemoglobin decreased (90 g/L), creatine kinase was 1258 U/L, creatine kinase isoenzyme was 40.2 U/L, pituitary function showed hypothyroidism, insulin-like growth factor decreased, glycosylated hemoglobin was 11.7%, blood lactic acid was 5.81 mmol/L, homocysteine was 20.998 µmol/L. The antibody against paraneoplastic neurological syndrome was negative. Autoimmune thyroid antibody, antinuclear antibody spectrum, antineutrophil cytoplasmic antibodies, anti-double-stranded DNA antibody, anticardiolipin antibody, immunoglobulin, anti-streptococcal hemolysin, compliment, and tumor markers were not significantly abnormal.

**Figure 3.**
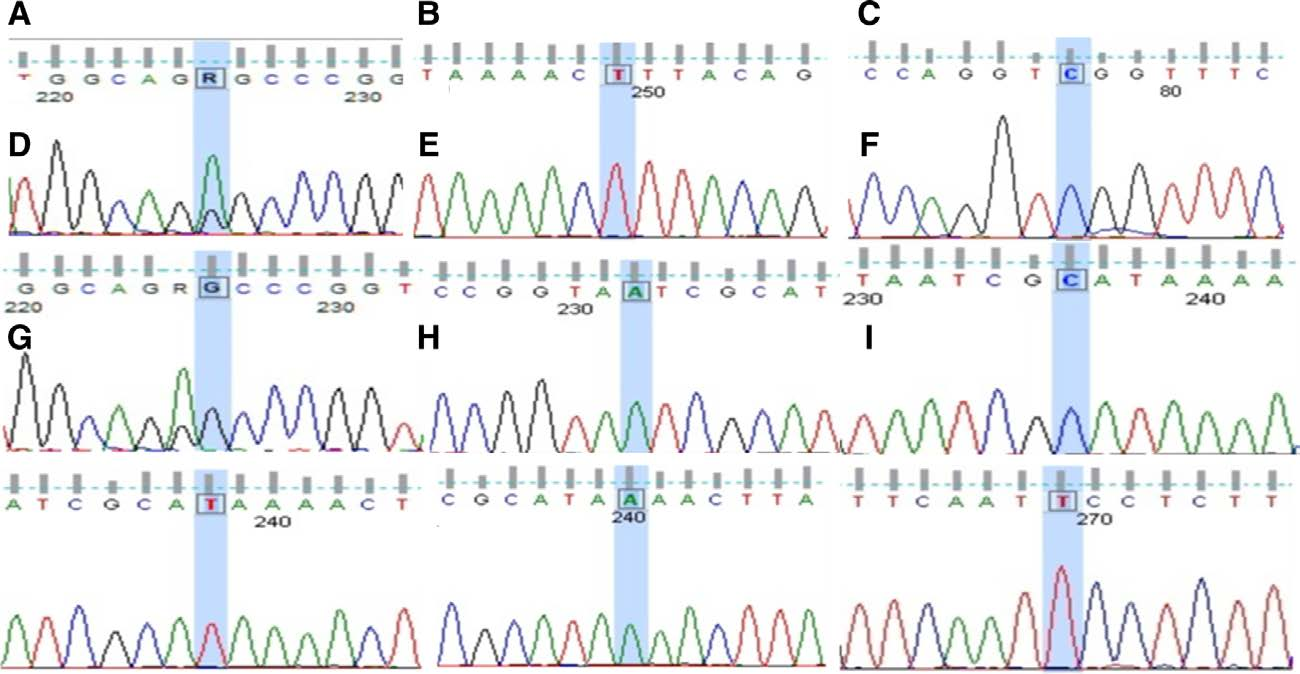
MtDNA gene associated with MELAS syndrome. MELAS = mitochondrial encephalopathy, lactic acidosis and stroke-like episodes, mtDNA = mitochondrial deoxyribonucleic acid.

**Figure 4.**
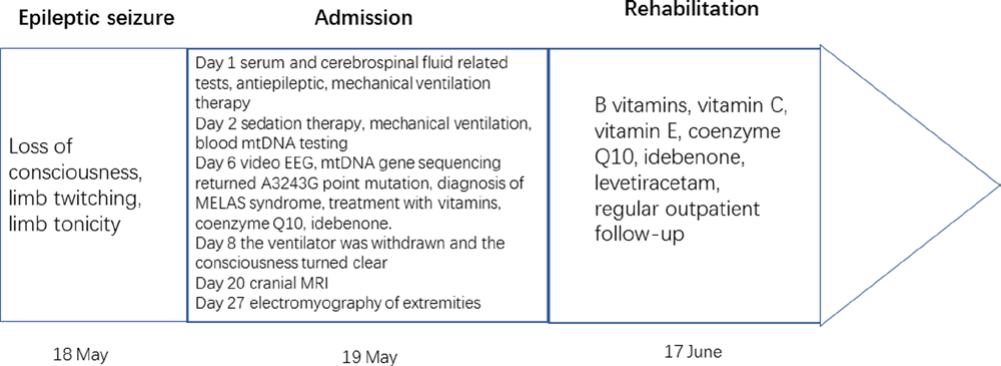
Timeline of clinical events, diagnostic-therapeutic approach, and clinical outcome.

Considered the diagnosis of symptomatic epilepsy, the patient was treated with mechanical ventilation, midazolam, and levetiracetam, and the limb twitching symptoms were controlled, but clinical, subclinical epileptiform discharges were present. The patient was comatose, chronically bedridden, with gastrointestinal dysfunction, and was treated prophylactically with antibiotics against infection, parenteral nutrition, and other supportive measures. Electroencephalography, cranial magnetic resonance imaging, autoimmune-related antibodies, electromyography, mtDNA, fundus, pure tone audiometry, cognitive function, and other related tests were completed. mtDNA gene sequencing returned A3243G point mutation after 6 days. Other regions associated with MELAS syndrome, including 8 common pathogenic mutations, were not found (Fig. 3), and MELAS syndrome was clearly diagnosed, B vitamins, vitamin C, vitamin E, coenzyme Q10, and idebenone were given, and mechanical ventilation and midazolam were stopped after 8 days. He was discharged from the hospital on 30 days and continued symptomatic treatment with B-vitamins, vitamin C, vitamin E, coenzyme Q10, and idebenone, and antiepileptic treatment with levetiracetam, with outpatient follow-up. Figure 4 shows a timeline of the clinical milestones of the patient.

## 3. Discussion

Our team reports a case of MELAS syndrome with a non-stroke-like onset and diffuse posterior white matter lesions. Our patient is a middle-aged woman. Her main symptoms are seizures, hearing loss, and gastrointestinal dysfunction. Patients diagnosed with MELAS syndrome based on A3243G point mutation found by mtDNA gene sequencing.^[7]^

It was previously thought that the majority of inherited cerebral white matter lesions were lysosomal storage disorders or peroxisomal disorders. However, in recent years, cerebral white matter lesions have been increasingly recognized as a common feature in patients with mitochondrial disorders.^[3–5]^ This may be caused by molecular defects in the mitochondrial genome or in nuclear genes. It is first realized in classical mitochondrial syndromes associated with mutations in mtDNA, such as mitochondrial encephalomyopathy with MELAS, Leigh disease, and Kearns-Sayre syndrome. Deficiencies in respiratory chain complexes I, II, IV, and V usually lead to Leigh disease; most of them are due to defects in nuclear genes that may lead to severe early-onset cerebral white matter lesion.^[3,8]^ With the recent discovery of nuclear genes responsible for defects in the mitochondrial respiratory chain, Leigh disease with white matter involvement caused by nuclear genes encoding complex subunits and assembly factors has been reported. In addition, cerebral white matter lesions in mitochondrial syndrome are caused by a set of nuclear genes involved in maintaining the integrity of mtDNA, such as DNA polymerase gamma, and thymidine phosphorylase and MPV17 defects have also been reported.^[3,9–12]^ Mutations in mitochondrial aspartyl-tRNA synthetase have been found to be associated with white matter lesions in the brainstem and spinal cord.^[13,14]^ The characteristics of cerebral white matter lesions may depend on the molecular pathology of mitochondrial disease. In general, lesions caused by mtDNA point mutations predominantly affect the deep gray matter, with less involvement of the peripheral white matter. Diffuse supratentorial, most likely necrotizing white matter encephalopathy, involving the deep white matter, is more frequently observed in patients with Leigh disease caused by mutations in nuclear genes encoding complex I or IV subunits and assembly factors.^[3–5]^

Mitochondrial encephalomyopathy with MELAS is the most common maternally inherited multisystem disorder. Patients with MELAS often have short stature, and the main symptoms include generalized tonic-clonic seizures, recurrent migraine, muscle weakness or motor intolerance, and sensorineural hearing loss. Lactic acidosis and stroke-like episodes with hemiparesis and cortical blindness are characteristic. Early development is usually slow. Clinical manifestations may occur in childhood or early adulthood. Molecular defects occur in mtDNA mutation hotspots, namely the tRNA Leu (UUR) gene.^[15,16]^ The A3243G mutation in mtDNA accounts for 80% of MELAS syndrome. Mutations in other tRNA and mRNA genes causing MELAS syndrome have also been observed. Electron microscopy showed mitochondrial hyperplasia as an enlargement or increase in mitochondria, characteristic of tRNA mutations in mtDNA. Serrated red fibers in muscle biopsies are common in older patients but are usually not evident in young children.^[3]^ Magnetic resonance imaging of the brain shows infarct-like lesions mainly affecting the occipital cortex, which usually do not correspond to a vascular distribution. Small nonspecific periventricular white matter lesions may also occur.^[17]^ In addition to stroke-like lesions, about half of the patients with MELAS reported white matter lesions in the brain.^[6]^ White matter lesions of the brain in MELAS are usually asymmetrical in the occipital, temporal, or frontal lobes of the subcortex.^[18]^ Patients may show diffuse gliosis in the white matter of the brain and cerebellum,^[19]^ and myelin loss and fibromatosis in the subcortical and deep white matter may be seen on brain computed tomography.^[20]^ Neuropathological studies may show multiple foci of old and new necrosis in the brain and cerebellum.^[21,22]^ However, diffuse symmetrical posterior cerebral white matter is very rare.^[23]^

Diffuse symmetric posterior cerebral white matter lesions are extremely rare in MELAS syndrome with m3243A>G mutations and lack of stroke-like episodes, and their pathophysiology is unknown. Currently, there are very few reports in the national and international literature.^[23]^ Our patient’s brain lesions were predominantly posterior, and although nonspecific, mtDNA gene sequencing confirmed the diagnosis of MELAS syndrome.

## 4. Conclusion

MELAS syndrome without stroke-like episodes of diffuse posterior cerebral white matter lesions is rare in clinical practice, and the possibility of MELAS syndrome should be considered in symmetric posterior cerebral white matter lesions. Unknown causes of white matter encephalopathy, short stature, body surface hypertrichosis, hyperlactatemia combined with deafness, and diabetes mellitus are to be thought of as a possible mitochondrial diseases. Early recognition, as well as effective symptomatic treatment, may improve the prognosis.

## Acknowledgments

The authors thank the patient for his participation in this study.

## Author contributions

**Conceptualization:** Peng Bai.

**Data curation:** Peng Bai, Yinling Feng, Jin Chen.

**Funding acquisition:** Peng Bai.

**Writing – original draft:** Peng Bai.

**Writing – review & editing:** Peng Bai, Yinling Feng, Jin Chen, Hong Chang.
